# Stoichiometric and Catalytic Synthesis of Alkynylphosphines

**DOI:** 10.3390/molecules171214573

**Published:** 2012-12-07

**Authors:** Elise Bernoud, Romain Veillard, Carole Alayrac, Annie-Claude Gaumont

**Affiliations:** Laboratoire de Chimie Moléculaire et Thioorganique, UMR CNRS 6507, INC3M FR3038, ENSICAEN & Université de Caen Basse-Normandie, 14050 Caen, France; E-Mails: e.bernoud@gmail.com (E.B.); romain.veillard@ensicaen.fr (R.V.)

**Keywords:** organophosphorus chemistry, phosphines, phosphine-boranes, homogeneous catalysis

## Abstract

Alkynylphosphines or their borane complexes are available either through C–P bond forming reactions or through modification of the phosphorus or the alkynyl function of various alkynyl phosphorus derivatives. The latter strategy, and in particular the one involving phosphoryl reduction by alanes or silanes, is the method of choice for preparing primary and secondary alkynylphosphines, while the former strategy is usually employed for the synthesis of tertiary alkynylphosphines or their borane complexes. The classical C–P bond forming methods rely on the reaction between halophosphines or their borane complexes with terminal acetylenes in the presence of a stoichiometric amount of organometallic bases, which precludes the access to alkynylphosphines bearing sensitive functional groups. In less than a decade, efficient catalytic procedures, mostly involving copper complexes and either an electrophilic or a nucleophilic phosphorus reagent, have emerged. By proceeding under mild conditions, these new methods have allowed a significant broadening of the substituent scope and structure complexity.

## 1. Introduction

Alkynylphosphines are relevant versatile compounds due to the presence of two important functions, a phosphorus atom—a key-atom in metal coordination [1]—and an alkynyl moiety, which can also coordinate to metals and is known to display a rich and diverse chemistry [2].

In the past, these phosphines were mainly used in coordination chemistry for the assembly of various mono- or bi-metallic complexes and clusters [3], by taking advantage of their diverse coordination modes to transition metals, via the phosphorus atom and/or the triple bond. It is noteworthy that the range of alkynylphosphines involved in those complexes and clusters is almost exclusively covered by diphenylphosphino-phenylacetylene and bis(diphenylphosphino)acetylene.

It is only recently that the high potential of alkynylphosphines as ligands for homogeneous catalysis has emerged through a few relevant catalytic applications [4]. Notably P-stereogenic alkynylphosphines were shown to induce excellent enantioselectivity in several asymmetric transition metal-catalyzed reactions [5]. Moreover the easy transformation of alkynylphosphines into valuable functionalized alkenyl mono- or di-phosphines through addition reactions on the triple bond was recently demonstrated [6]. These new applications as ligands for homogeneous catalysis and building blocks for organophosphorus chemistry have aroused the need for new general synthetic methods allowing the access to a broad substitution pattern.

**Scheme 1:**
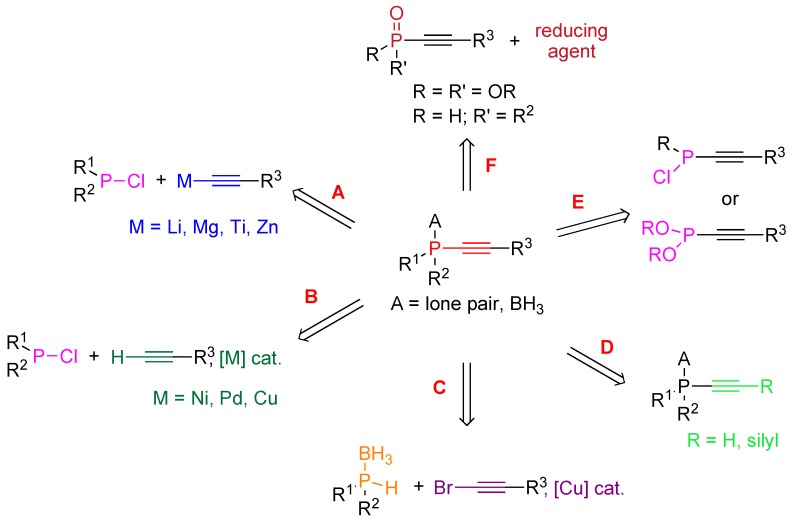
Strategies A–F for the synthesis of alkynylphosphines and/or their borane complexes.

The classical method for preparing alkynylphosphines is based on the nucleophilic substitution reaction at the phosphorus atom of a halophosphine by a metal (lithium, magnesium, titanium or zinc) acetylide at low temperature (Scheme 1, path A). This methodology was recently successfully applied to the synthesis of a few enantioenriched P-stereogenic alkynylphosphine-boranes. The reaction however suffers from severe limitations due to the weak configurational stability of halophosphines.

Catalytic variants of this reaction have been recently described as useful alternative approaches when sensitive functional groups are present (Scheme 1, path B). They rely on the cross-coupling reactions of terminal alkynes with chlorophosphines in the presence of a catalytic amount of nickel, palladium or copper complexes. Both stoichiometric and catalytic methods are mainly based on an electrophilic phosphorus derivative—a halophosphine—as starting material. The use of a nucleophilic phosphorus derivative has been only little explored so far. In the stoichiometric series, attempts involving treatment of alkynylphosphonium chlorides with a base failed to afford the expected tertiary alkynylphosphines because their oxidation could not be avoided under the reaction conditions [7]. On the other hand the catalytic synthesis of tertiary alkynylphosphine-boranes based on the copper(I)-catalyzed cross-coupling reaction between secondary phosphine-boranes and bromoalkynes was shown to proceed readily (Scheme 1, path C). A further strategy towards alkynylphosphines relies on the transformation of alkynylphosphorus derivatives through modification of the alkyne (Scheme 1, path D) or the phosphorus function (Scheme 1, paths E and F). Notably the reduction of the phosphoryl moiety of alkynylphosphorus derivatives (Scheme 1, path F) is highly relevant for the synthesis of primary and secondary alkynylphosphines, which are air- and heat-sensitive compounds.

The scope of this review encompasses the synthesis of alkynylphosphines and their borane complexes [8]. The stoichiometric methods for C–P bond formation will be first surveyed followed by the catalytic procedures. Finally the methods based on the transformation of alkynylphosphorus derivatives will be detailed.

## 2. Nucleophilic Substitution at P-atom of Halophosphines

Tertiary alkynylphosphines are classically prepared through the reaction of chlorophosphines and metal acetylides. For secondary alkynylphosphines, only those bearing sterically encumbered substituents are available through this procedure. 

### 2.1. Synthesis of Secondary Alkynylphosphines

**Scheme 2:**
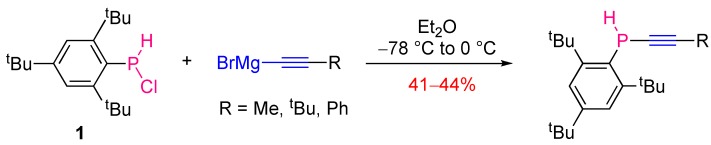
Synthesis of secondary alkynylphosphines via nucleophilic substitution.

Märkl and Reitinger reported the synthesis of a set of secondary alkynylphosphines based on the reaction of the bulky secondary chlorophosphine **1** with various alkyne Grignard reagents in diethyl ether at low temperature [9] (Scheme 2).

**Scheme 3:**
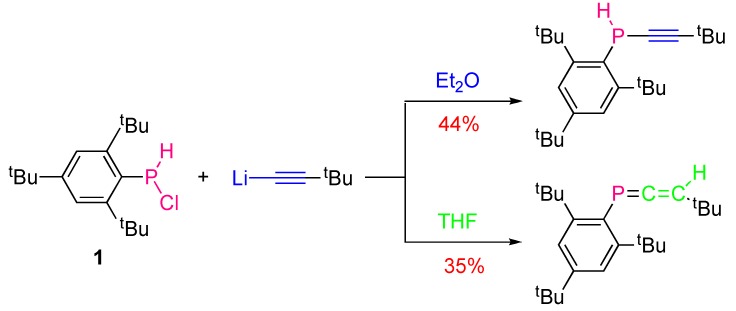
Solvent-effect on the outcome of the reaction of lithium acetylides with a secondary chlorophosphine.

The analogous reactions of chlorophosphine **1** with lithium acetylides in THF did not afford alkynylphosphines but their 3*H*-phosphaallene tautomers, because of the stronger basicity of lithium acetylides in comparison to the related Grignard reagents. One exception was found with lithium trimethylsilylacetylide, which led to the alkynyl product under the same conditions. The reaction of secondary chlorophosphine **1** with metal acetylides proved to be not only metal- and substrate- but also solvent-dependent, probably because of the importance of the acetylide solvation. For example the reaction of **1** with lithium *tert*-butylacetylide led to the alkynyl product in diethyl ether and to the allenyl product in THF (Scheme 3).

### 2.2. Synthesis of Tertiary Alkynylphosphines

#### 2.2.1. Version with Achiral Reagents

**Scheme 4:**
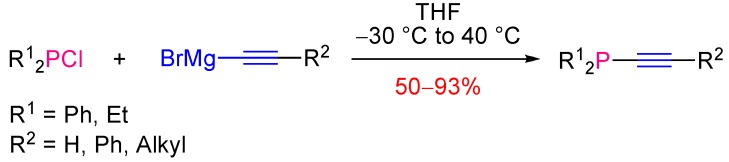
Reaction of magnesium acetylides with chlorophosphines towards alkynylphosphines.

The oldest methods for the preparation of tertiary alkynylphosphines are based on the nucleophilic substitution reaction at the phosphorus atom of halophosphines by lithium [10] or magnesium [11] acetylides. For example, Cadiot and Chodkiewicz described the synthesis of a set of alkynylphosphines in good to excellent yields (50%–93%) through the reaction of Grignard reagents and dialkyl- or diarylchlorophosphines in THF at −30 °C to 40 °C (Scheme 4).

**Scheme 5:**
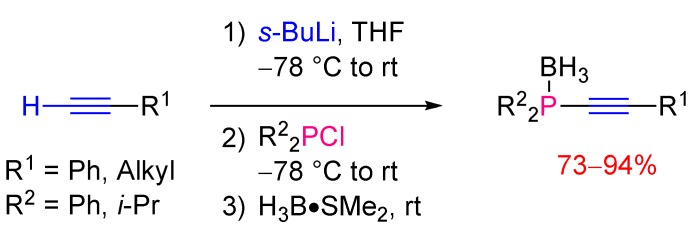
Synthesis of alkynylphosphine-borane complexes from lithium acetylides.

These procedures involving magnesium or lithium acetylides are still commonly used nowadays. For example Montchamp [12] described the synthesis of several alkynylphosphines using *sec*-butyllithium to generate *in situ* the lithium acetylide reagent. Subsequent treatment with BH_3_•SMe_2_ afforded the corresponding borane adducts in good yields (Scheme 5).

**Scheme 6:**
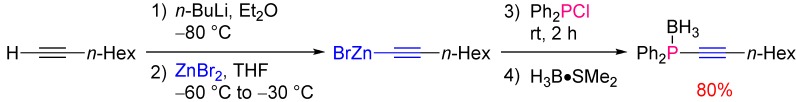
Synthesis of alkynylphosphines using a zinc acetylide.

Besides magnesium and lithium reagents, copper- [13], tin- [14], or silver- [15] acetylides have been involved in a few examples. In view of their high reactivity, magnesium and lithium acetylides are suitable for the formation of alkynylphosphines which do not bear functional groups sensitive to nucleophilic attack such as esters. To prevent the occurrence of side reactions, the use of more selective organometallic reagents like zinc [16] and titanium [17] acetylides was explored. Knochel demonstrated that zinc organometallics were effective for the synthesis of polyfunctionalized phosphines and described one example in the alkynyl series, but on a simple substrate. The alkynylzinc bromide reagent resulting from the transmetalation of 1-hexynyllithium with zinc bromide reacted with chlorodiphenylphosphine to provide the corresponding alkynylphosphine-borane in 80% yield after complexation with borane (Scheme 6).

**Scheme 7:**
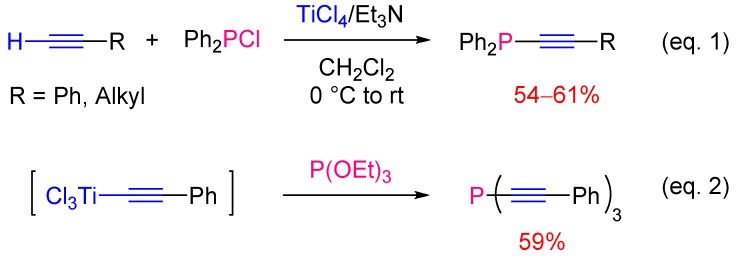
Synthesis of alkynylphosphines using titanium acetylides.

Periasamy [17] reported the synthesis of alkynylphosphines using alkynyl titanium reagents. The latter were not obtained by transmetalation of alkynyllithium reagents [18], but were generated *in situ* by direct metalation of 1-alkynes with the combination of titanium tetrachloride and triethylamine in dichloromethane (Scheme 7, Equation 1). Alkyne dimerisation may occur as a side reaction. For example, the reaction of phenyl acetylene with diphenylchlorophosphine afforded the corresponding alkynylphosphine in 54% yield, together with 2% of the diyne. Not only diphenylchlorophosphine but also triethylphosphite could serve as an electrophile. The substitution of all ethoxy groups was observed leading to tris(phenylethynyl)phosphine in 59% yield (Scheme 7, Equation 2).

#### 2.2.2. Asymmetric Version

**Scheme 8:**
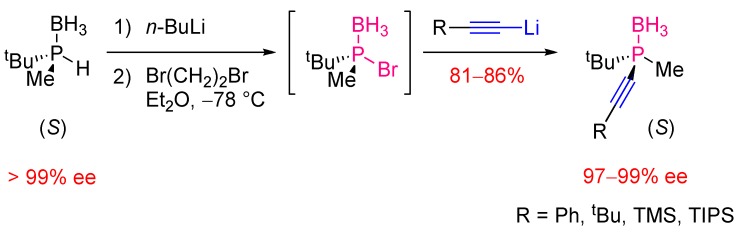
Synthesis of enantioenriched P-stereogenic alkynylphosphine-boranes.

The single example relating the synthesis of a set of enantiopure P-stereogenic alkynylphosphines was described by Imamoto [5] (Scheme 8). The procedure relies on the *in situ* generation of a P-stereogenic bromophosphine-borane from an enantiopure secondary phosphine-borane via deprotonation by *n*-butyllithium in diethyl ether at −78 °C and subsequent halogenation of the resulting lithium phosphido-borane by treatment with dibromoethane. The bromophosphine-borane was not isolated because of its tendency to racemize at room temperature, but was treated *in situ* with a set of alkynyl lithium reagents. The nucleophilic substitution reaction was shown to occur with clean inversion of configuration at the phosphorus atom, delivering the alkynylphosphines in high yields and excellent stereospecificity.

The procedure is up to now only applicable to enantiopure *tert*-butylmethylbromophosphine-borane, which displays a sufficient configurational stability at low temperature. This successful example is probably difficult to extend to a wide variety of phosphines due to the weak configurational stability of halophosphine-boranes.

## 3. Catalytic C-P bond Forming Reactions

Within the last ten years a few catalytic approaches towards alkynylphosphines have emerged. They rely on the use of either (i) electrophilic phosphorus derivatives, using nickel, palladium or copper complexes as catalytic system, or (ii) nucleophilic phosphorus derivatives using a copper(I) complex.

### 3.1. Electrophilic Phosphorus Reagent

**Scheme 9:**
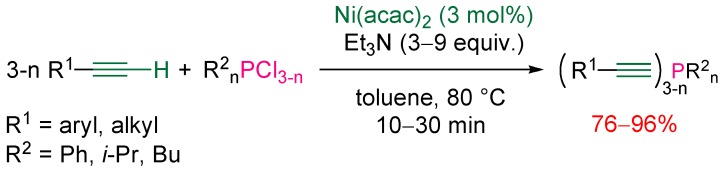
Nickel-catalyzed cross-coupling of terminal alkynes with chlorophosphines.

As previously mentioned, alkynylphosphines are classically available through the reaction of chlorophosphines with terminal alkynes in the presence of stoichiometric amounts of organometallic bases. The Beletskaya group reported in 2003 [19] a catalytic variant, which was based on the use of nickel or palladium complexes in the presence of an excess of triethylamine. Nickel complexes were shown to display a higher activity than the palladium ones, the best catalysts being Ni(cod)_2_ and Ni(acac)_2_. The reaction with aryl or alkyl acetylenes readily proceeded in toluene at 80 °C for 10 to 30 min or at room temperature for 6 to 10 h (Scheme 9). It is noteworthy that the selective monosubstitution of one chloride atom from di- or tri-chlorosubstrates is not possible.

**Scheme 10:**
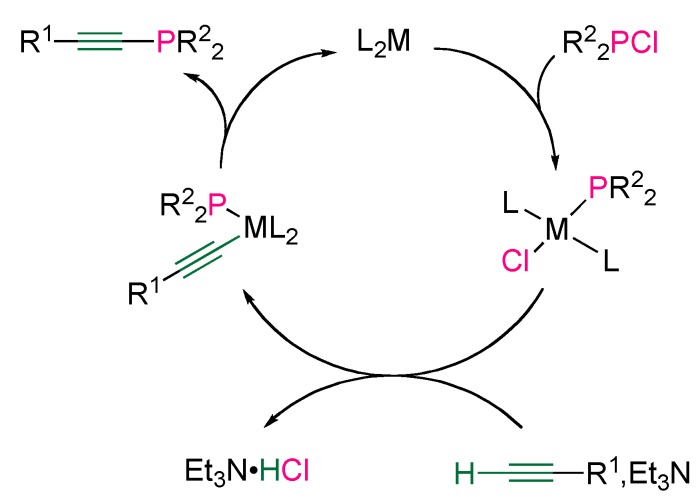
Simplified postulated mechanism of nickel- or palladium-catalyzed cross-coupling between terminal alkynes and chlorophosphines.

The simplified postulated mechanism first involves the insertion of the metal into the P–Cl bond of the chlorophosphine. The resulting metal phosphido complex undergoes transmetalation with the *in situ* formed acetylide ion, followed by reductive elimination (Scheme 10).

**Figure 1:**
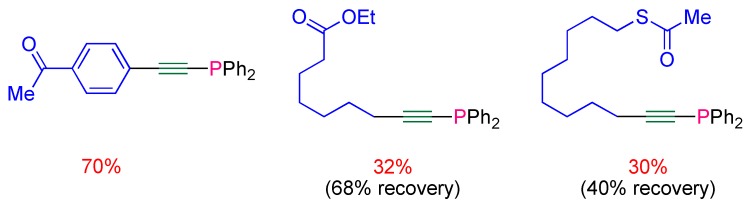
Functionalized alkynylphosphines synthesized via the nickel-catalyzed procedure.

The procedure was applied to the synthesis of a set of functionalized alkynylphosphines bearing carbonyl groups, which would have been difficult to access via the stoichiometric method (Figure 1). However in the case of aliphatic alkynes, a full conversion could not be reached and poor yields (around 30%) were obtained [20].

**Scheme 11:**
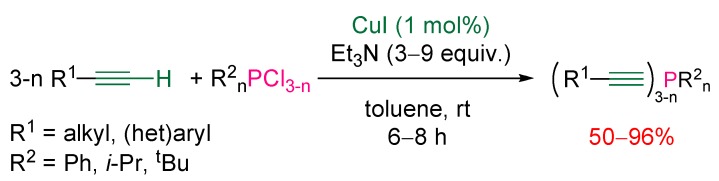
Copper-catalyzed cross-coupling of terminal alkynes with chlorophosphines.

Another limitation of this nickel-catalyzed procedure was its lack of efficiency with substrates bearing an alkoxy or amino group. To overcome these limitations a more versatile catalytic method was developed by the same authors. The method is based on the use of copper salts [21] and proceeds under mild conditions. Copper(I) iodide was used as the copper source in the presence of an excess of triethylamine in toluene at room temperature (Scheme 11). The reaction scope is broad and encompasses alkyl and (het)aryl acetylenes. Regarding the phosphorus coupling partner, aryl and alkyl chlorophosphines are suitable substrates.

**Scheme 12:**
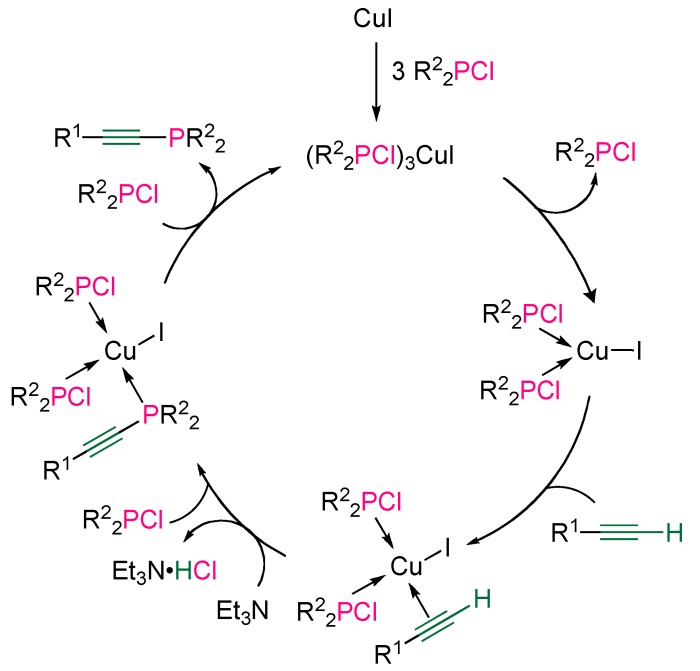
Postulated mechanism of copper-catalyzed cross-coupling between terminal alkynes and chlorophosphines.

The postulated mechanism does not rely on P–Cl bond activation by the metal, as proposed in the case of Ni or Pd catalysis, but copper π-complexation of the terminal alkyne, followed by nucleophilic substitution at the phosphorus atom of the chlorophosphine in the presence of triethylamine, as outlined on Scheme 12. This hypothesis was supported by the isolation of the tetrahedral complex (Ph_2_PCl)_3_CuI, which is the first active species involved in the suggested catalytic cycle.

**Scheme 13:**
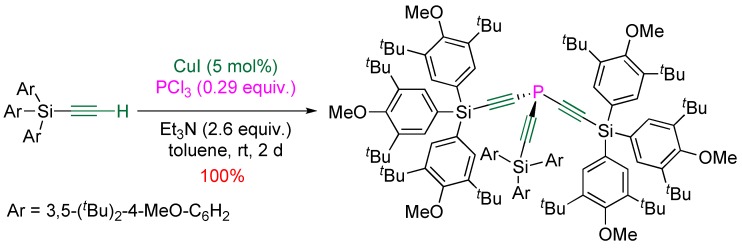
Copper(I)-catalyzed synthesis of functionalized triethynylphosphines.

This general and mild procedure was successfully applied to the synthesis of bulky triethynylphosphine ligands [22,23] (Scheme 13).

### 3.2. Nucleophilic Phosphorus Reagent

**Scheme 14:**
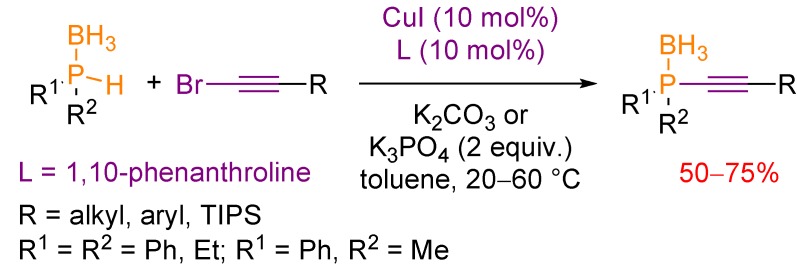
Copper(I)-catalyzed reaction of secondary phosphine-boranes with 1-bromoalkynes towards alkynylphosphine-boranes.

The Gaumont group reported the only example of a catalytic synthesis of alkynylphosphines, relying on nucleophilic phosphorus reagents [24]. The alkynylphosphines were obtained as their borane complexes. The methodology is based on the copper-catalyzed cross-coupling of secondary phosphine-boranes with alkyl or aryl 1-bromoalkynes using CuI/1,10-phenanthroline as catalytic source (Scheme 14). The reaction proceeds in toluene under mild conditions (20 to 60 °C, weak base).

**Scheme 15:**
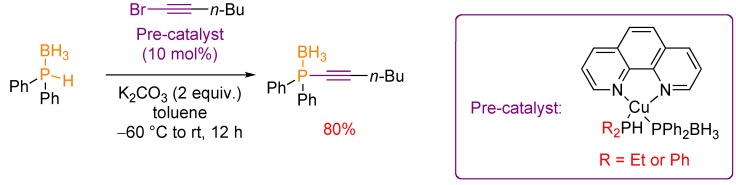
P-alkynylation of secondary phosphine-boranes catalyzed by well-defined copper(I) phosphido-borane complexes.

Preliminary mechanistic studies are consistent with the involvement of a copper(I) phosphido-borane complex as the reactive species. Indeed neutral copper phosphido-boranes [R_2_PHCuPPh_2_BH_3_phen], which could be isolated for the first time and fully characterized via experimental and computational methods [25], were shown to serve as pre-catalysts in the P-alkynylation of secondary phosphine-boranes with 1-bromoalkynes (Scheme 15). 

**Scheme 16:**
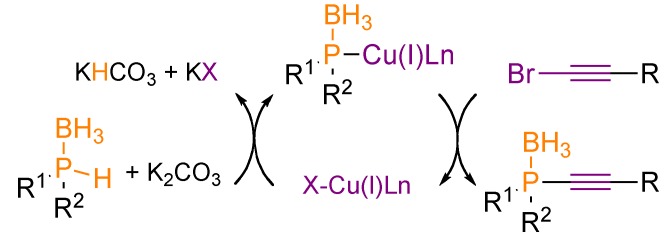
Putative mechanistic cycle for the copper(I)-catalyzed P-alkynylation of secondary phosphine-boranes.

The putative mechanism of the reaction involves oxidative addition of the copper(I) phosphido-borane complex to the 1-bromoalkyne, followed by reductive elimination, leading to the cross-coupling product and allowing the release of the copper(I) catalyst (Scheme 16).

## 4. Transformation of Alkynylphosphorus Derivatives

Regarding the synthesis of tertiary alkynylphosphines, modification of the alkynyl or the phosphorus function of alkynyl phosphorus derivatives is a less commonly used method in comparison to C–P bond formation. On the other hand, in the case of secondary and primary alkynylphosphines, the reduction of the phosphoryl moiety of phosphine oxides and phosphonates respectively, is the method of choice for their preparation.

### 4.1. Functionalization of the Triple bond Moiety

**Scheme 17:**
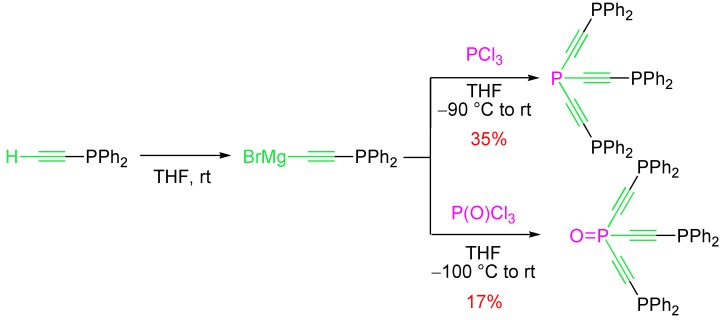
Metalation and functionalization of ethynylphosphine.

Classical alkyne chemistry has been applied in the functionalization of alkynylphosphines through their metalation and subsequent reaction with an electrophile. With the aim of preparing dendrimers, Caminade and Majoral [26] generated the Grignard reagent of ethynylphosphine as building block for the synthesis of mono-, di-, tri-, and tetra-phosphines (Scheme 17). It is noteworthy that the lithium acetylide of ethynylphosphine was not suitable for these transformations, complex mixtures being formed in this case.

**Scheme 18:**
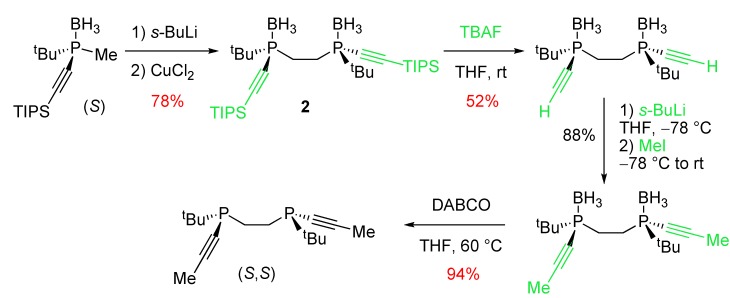
Synthesis of an alkynyl analogue of DIPAMP.

The Imamoto group [5] described a 4-step reaction sequence allowing the synthesis of an alkynyl variant of BisP* ligands, analogous to DIPAMP [27], from an enantiopure silyl alkynylphosphine-borane (Scheme 18). The first step was a dimerization reaction through copper(II)-catalyzed oxidative coupling leading to the bis-alkynylphosphine-borane **2**. The next step was the silyl deprotection by treatment with TBAF. It was followed by deprotonation with *sec*-BuLi and methylation of the resulting lithium derivative with methyl iodide. Finally borane removal by treatment with DABCO (1,4-diazabicyclo[2.2.2]octane) afforded the bis-alkynylphosphine. 

### 4.2. Nucleophilic Substitution at Phosphorus Atom

**Scheme 19:**
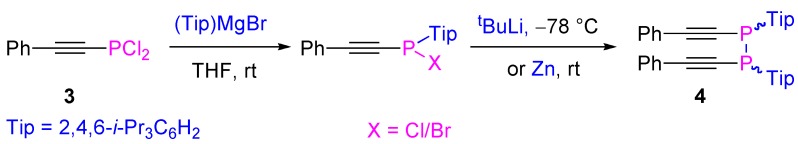
Synthesis of alkynylphosphines from alkynyl-chlorophosphines.

The nucleophilic substitution at the phosphorus atom of alkynylchlorophosphines by Grignard reagents has been used for the building of complex structures. As an example, the Yoshifuji group [28] described the synthesis of the dialkynyldiphosphine **4** from alkynyldichlorophosphine **3** (Scheme 19).

**Scheme 20:**
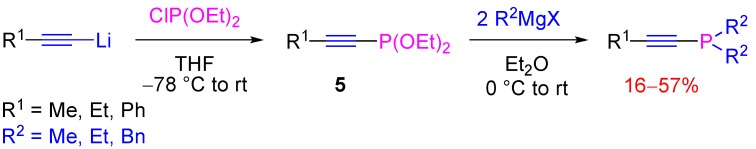
Synthesis of alkynylphosphines from *in situ* generated alkynylphosphonites.

Besides chlorophosphines, phosphonites can serve as precursors of alkynylphosphines. The Aguiar group [29] reported the synthesis of a set of alkynylphosphines through the reaction of the *in situ* generated diethyl alkynyl-1-phosphonite **5** and Grignard reagents (Scheme 20).

### 4.3 Reduction of the Phosphoryl Moiety

As described in the Section 2.1, a few secondary alkynylphosphines were prepared via the reaction of chlorophosphines and metal acetylides. However it concerned specific substrates stabilized by the presence of bulky substituents on the phosphorus atom. 

**Scheme 21:**
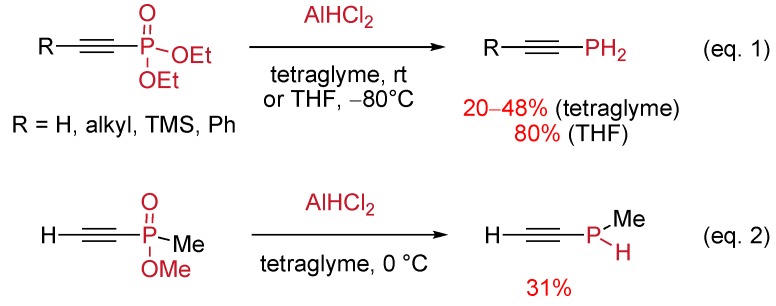
Synthesis of primary and secondary alkynylphosphines via phosphoryl reduction by alanes.

A more straightforward and general access to unstabilized primary and secondary alkynylphosphines relies on the reduction of phosphoryl derivatives by alanes or silanes [30]. Guillemin and Denis [31] developed the chemoselective reduction of alkynylphosphonates using AlHCl_2_ as reducing agent. The best yields were obtained when the reaction was performed at low temperature in diethyl ether or THF (Scheme 21, Equation 1). The same alane reducing agent was less suitable for the preparation of secondary alkynylphosphines from alkynylphosphinates. Indeed C–P bond cleavage occurred as a side-reaction [32], thus lowering the yields (Scheme 21, Equation 2).

**Scheme 22:**
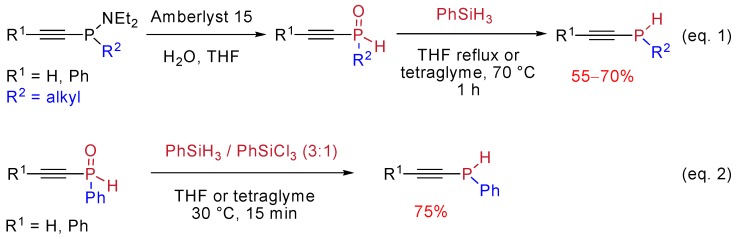
Synthesis of secondary alkynylphosphines via phosphoryl reduction by silanes.

A mild and practical synthesis of secondary alkynylphosphines was reported by Gaumont and Denis [33]. It is based on the reduction of secondary alkynylphosphine oxides in the presence of silane derivatives. The starting phosphine oxides were available through acidic cleavage of aminophosphine precursors. The appropriate silane reducing agent depended on the substituents at the phosphorus atom. Phenylsilane was used as reducing agent in the case of P-alkyl derivatives (Scheme 22, Equation 1) while the reduction of P-phenyl derivatives was only partial under these conditions. It was finally achieved with the combination of phenylsilane and trichlorophenylsilane in a 3:1 ratio (Scheme 22, Equation 2). The active reducing agent is believed to be the *in situ* generated PhSiHCl_2_. The presence of chlorine atoms on silicon, by increasing the oxophilic properties of the silane reagent, would explain its higher reactivity.

## 5. Conclusions

The synthetic pathways towards alkynylphosphines used to be restricted to stoichiometric procedures involving the use of organometallic bases, which limited the scope of the substitution pattern to robust functional groups. Since less than a decade, benefiting from the renaissance of copper catalysis for C–C and C–Heteroatom bond forming reactions, catalytic procedures have emerged, which involve either an electrophilic or a nucleophilic phosphorus derivative. By proceeding under mild conditions, these new catalytic methods have broadened the substituent scope and have been successfully applied to the synthesis of functionalized complex phosphine structures.

Future developments comprise catalytic procedures avoiding the use of halogen-containing derivatives, such as oxidative cross-couplings, which have proven their efficiency for the synthesis of alkynylphosphonates [34], but whose application to the synthesis of alkynylphosphines is challenging because of the high sensitivity of phosphines towards oxidation. Another challenge is the extension to the asymmetric series through the use of chiral ligands associated with the metal catalyst. 
